# A computerized red glass test for quantifying diplopia

**DOI:** 10.1186/s12886-017-0465-8

**Published:** 2017-05-17

**Authors:** Han Soo Yoo, Eunjeong Park, Soolienah Rhiu, Hyuk-Jae Chang, Kyoungsub Kim, Joonsang Yoo, Ji Hoe Heo, Hyo Suk Nam

**Affiliations:** 1 Department of Neurology, Yonsei University College of Medicine, 50 Yonsei-ro, Seodaemoon-gu, Seoul, 03722 Korea; 2 Cardiovascular Research Institute, Yonsei University College of Medicine, Seoul, Korea; 3 Health IT Research and Development Center, Yonsei University College of Medicine, Seoul, Korea; 4 Department of Ophthalmology, Dongtan Sacred Heart Hospital, Hallym University College of Medicine, Hwaseong, Korea; 5 Department of Cardiology, Yonsei University College of Medicine, Seoul, Korea

**Keywords:** Diplopia, Computer software, Neurologic examination, Quantification

## Abstract

**Background:**

Accurate evaluation of diplopia during bedside physical examination is challenging. We developed a new computerized red glass test (CRT) to detect, localize, and quantify diplopia and investigated whether the CRT is useful and feasible.

**Methods:**

During the CRT, a white dot randomly appears on a monitor. Because a red glass is applied on the right eye, a patient can see one white dot and one red dot when diplopia is present. We defined the degree of diplopia as the direct distance of the two points with the largest deviation and compared the degree with the Hess score and Hess area ratio.

**Results:**

We prospectively enrolled 14 patients with binocular diplopia. Test–retest reliability of the CRT was excellent (overall intraclass correlation coefficient = 0.948, 95% CI 0.939–0.956). The degree of diplopia in the CRT was well correlated with both the Hess score (*r* = 0.719, *p* = 0.005) and the Hess area ratio (*r* = −0.620, *p* = 0.018).

**Conclusions:**

The CRT can easily detect the presence of diplopia and provided the quantitative values of the degree of diplopia. The CRT was useful and feasible for improving routine bedside examination.

## Background

Diplopia is a subjective complaint of seeing two images of a viewed object. It can arise from neurologic, ocular, or extraocular muscle disorders. Monocular diplopia is usually caused by a disorder in one eye, whereas binocular diplopia arises from ocular misalignment caused by either neurologic or ophthalmic disorders [1]. Rapid and accurate evaluation of diplopia in a bedside examination is challenging. Popular bedside diplopia exams are the duction test, version test, the Maddox rod test, and the red glass test [2].

The red glass test is widely used in both emergency rooms and clinics because it can be performed quickly as an initial evaluation of diplopia [3]. However, the red glass test is not quantitative, and the results can vary test by test or between examiners. In contrast, objective and quantitative tests are the Hess screen test, the Lancaster red-green screen test, and the Lees screen test [4]. Although those tests are the gold standard for evaluation of diplopia, they require special devices and trained technicians. Because many acute stroke patients have neurological deficits that can cause falls, it might be risky to move them to the ophthalmic laboratories. In these patients, development of such healthcare applications will provide more opportunities to improve stroke management [5].

Therefore, we developed a new computerized red glass test (CRT) to assess patients with diplopia. This pilot study investigated whether it is useful and feasible as a bedside tool for evaluation of diplopia.

## Methods

### Development of a computerized diplopia test

**Fig. 1:**
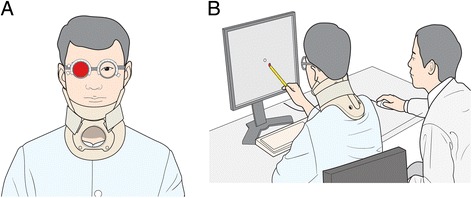
Performing the computerized red glass test (CRT). The CRT runs on any desktop computer with a color monitor larger than 19 in.. The subject sat on a chair in front of the monitor, aligned in the middle of monitor. A neck collar was applied to reduce head movement during the CRT. A red glass was applied to the right eye using an optical trial frame. Therefore, if ocular misalignment exists, the patient sees one white dot and one red dot (**a**). For accuracy and safety, a researcher helped clicking a mouse when the subjects indicated the red dot (**b**)

We developed a new software that runs on a desktop computer to detect, localize, and quantify diplopia. Although we used a 19-in. color monitor, any monitor or projector screen larger than 19 in. can be used. Each subject sat on a chair in front of the monitor, with his or her body aligned to the middle of the monitor. Room lights were dimmed to identify points more easily. An 8-mm white dot randomly appeared on nine cardinal positions of gaze. Since subtle head movements may prevent accurate measurement, a neck collar was applied. A red glass was applied on the right eye with an optical trial frame; therefore, patients saw one white dot and one red dot when they had ocular misalignment. To ensure accuracy and safety throughout the testing procedure, researchers assisted the patient by clicking a mouse whenever subjects indicated seeing a red dot with a stylus, as some patients could not maintain an upright posture after experiencing a stroke (Fig. 1).

We set the distance from the monitor to the patient as 40 cm. However, we made modifications according to the severity of diplopia. For patients with severe cases of diplopia, we set the distance to 30 cm, as the diplopic image could be out of range from the size of monitor. A distance below 30 cm was not applied and those patients were excluded from the study to avoid the effects of accommodation or vergence [6]. We adjusted the distance to 50 cm for the patients with very mild diplopia because the distance between white dot and red dot was too close to measure. As the Hess test, the interval between one white dot and another nearby white dot was automatically set to maintain 15° from the mid-point between the monitor and the patient. For example, when a patient sat 30 cm in front of the monitor, the interval between the two white dots was 30 cm x tangent of 15 degrees ≈ 8 cm. In the same manners, for distances of 40 cm and 50 cm, the intervals were 10.7 cm and 13.4 cm, respectively. Due to different distances between the monitor and the patient, we normalized our data using the same distance of 40 cm. To do that, the result for 30 cm distance was multiplied by 4/3, and the result for 50 cm distance was multiplied by 4/5.

The first screen of the CRT contained clinical information of patients, which consisted of their onset time, types of diplopia, as well as history of medico-surgical procedures, ophthalmologic diseases, and neurological diseases. The patients’ accompanying symptoms, signs, or neurologic deficits were also collected. For calibration, a researcher measured and inputted the distance between the patient and the monitor. Before the main CRT test, a practice session was provided. During the practice session, the subject learned how to perform the CRT. A main test began after the practice session. Subject repeated the CRT tests three times, which took about 3 min to complete.

The CRT program instantly recorded and calculated the absolute horizontal deviation and vertical deviation of red dot, as well as the direct distance from the red dot to the center dot after completion of the three trials. All data were saved automatically in an Excel worksheet (Microsoft Inc., Seattle, WA, USA). We defined the degree of CRT as the maximum direct distance between the two points. Therefore, a high score indicates severe diplopia. Along with quantitative data, the program immediately provided a figure showing the red and white dots in all the nine cardinal positions of gaze. This figure is similar with the report of the conventional red glass test. Using the summarized figure of CRT, an examiner could easily identify the presence of diplopia and estimate the restriction of the eye movements.

### Study subjects

In this pilot study, we prospectively enrolled consecutive patients with diplopia. To be enrolled, patients should have binocular diplopia, a visual acuity of more than 20/100, and normal retinal correspondence. Retinal correspondence was determined by a strabismologist (S. Rhiu) using the Bagolini striated glasses test. Exclusion criteria were the patients with very severe diplopia which cannot be measured with the CRT, inabilities to perform the tests (e.g., altered consciousness, poor cooperation, inability to sit, and severe aphasia), a life-threatening medical or surgical condition, or severe degree of dementia. We also recruited healthy volunteers with no history of neurological or ophthalmological diseases. We confirmed they have normal physical and ophthalmological examination. The Severance Hospital Institutional Review Board approved this study, and we obtained informed consent from all patients and volunteers.

### Neurological and ophthalmological examinations

We took medical histories and performed neurological and ophthalmological examinations for all study subjects. Along with the past medical history, we identified onset and progression of diplopia, its exacerbating and relieving factors, and the other associated symptoms. A neurologist performed complete neurological examinations, including a mental status evaluation, cortical function tests, cranial nerve tests, motor and sensory tests, cerebellar function tests, and reflexes. Along with routine ophthalmological examinations, an ophthalmologist measured visual acuity and retinal correspondence. Enrolled patients underwent both the CRT and the Hess test. A neurologist interpreted the diplopia using the CRT, whereas an ophthalmologist independently interpreted the diplopia using the Hess test.

### The Hess test

A skilled technician carried out the Hess test. Briefly, the Hess test was performed on a 120 × 120 cm^2^ screen, which was 1 m from the subject. The subject sat down in front of the Hess screen and wore red-green goggles. The red lens on the right eye was the fixating eye. Because of the red-green goggles, the subjects could see only red makers on the screen with the right eye, whereas they could see only green makers through the left eye with the green lens. While wearing the goggles, the subject used a laser pointer to indicate where a green line appears on the center of the red marker. The technician recorded and outlined those points. The patient repeated the exam with the green glass before the right eye and conducted the same process. Thus, the test evaluated the movement of each eye by dissociating binocular vision using red and green filters [4].

The degree of diplopia in the Hess test was quantified using the Hess score and the Hess area ratio (HAR) [7, 8]. The Hess score provides both horizontal and vertical deviations and is calculated using the displacement of individual points from the center, inner, and outer zones. The deviations of 16 outer points (*S*_o_), inner points (*S*_i_), and the center point (*S*_c_) are summed, and each point is weighted with individual factors (*F*_o_, *F*_i_, and *F*_c_ were 1, 4 and 8, respectively). The Hess score is calculated according to the following formula: Hess Score = *F*_o_ *S*_o_ + *F*_i_ *S*_i_ + *F*_c_ *S*_c_. The HAR is the percentage of square area on the affected side compared to the healthy side. The HAR represents how the diplopic area is contracted and is calculated by measuring the length of the inter-horizontal plot and the inter-vertical plot on the 30-degree line of the Hess chart using the following equation: 100 × (*A* × *B*)/(*A*1 × *B*1) (%), where *A* is the affected side length between horizontal plots, *B* is the affected side length between vertical plots, *A*1 is the healthy side length between horizontal plots, and *B*1 is the healthy side length between vertical plots. Thus, high Hess score or low HAR indicate severe diplopia.

### Statistical analysis

We used SPSS software 18.0 for Windows (SPSS Inc., Chicago, IL, USA) for statistical analysis. Data distributions were determined using the Kolmogorov-Smirnov test. All parameters of the CRT were not normally distributed. We reported the descriptive statistics using the median and interquartile range (IQR). The Mann-Whitney U test and the Wilcoxon signed-rank test were used for comparison of medians, and the Spearman test was used for correlation analysis between parameters. We calculated the test-retest reliability using the intraclass correlation coefficient (ICC). A two-tailed *p*-value of less than 0.05 was considered significant.

## Results

### Subject characteristics

We prospectively enrolled 14 patients with binocular diplopia and 10 healthy controls. All patients completed both the CRT and the Hess test. They were 7 men and 7 women with a median age of 46 years old (IQR 29–62.25). On the bedside exams, 9 patients had vertical diplopia, and 5 patients had horizontal diplopia. The affected sides were 8 on the right side and 6 on the left side. The presumed causes of diplopia were 7 infranuclear palsies (50%), 2 supranuclear palsies (14.3%), 3 orbital disorders (2 traumatic and 1 myogenic) (21.4%), and 2 unknown (14.3%). The median time interval from symptom onset to evaluation was 30 days (IQR 12.75–62.5) (Table 1).

**Table 1:** Demographic and clinical characteristics of patients with diplopia

	Sex/age	Side	Type of diplopia	Interval from symptom onset to test	Lesion	Interpretation by the CRT	Interpretation by the Hess screen test	Concordance
1	M/48	Rt	Vertical	10D	Infranuclear	Oculomotor palsy	Oculomotor palsy	Concordant
2	M/65	Lt	Horizontal	6 M 1D	Infranuclear	Abducens palsy	Abducens palsy	Concordant
3	M/66	Rt	Vertical	1 M 8D	Idiopathic	SO palsy	SO palsy	Concordant
4	F/64	Lt	Vertical	30D	Orbital (traumatic)	SO palsy	SO palsy	Concordant
5	M/35	Lt	Horizontal	4D	Supranuclear	MR palsy	Adduction palsy	Concordant
6	F/53	Rt	Vertical	17D	Orbital (myogenic)	MR and IO palsy	Upward gaze palsy	Concordant
8	F/27	Lt	Vertical	1 M	Infranuclear	Trochlear palsy	Trochlear palsy	Concordant
9	M/38	Lt	Horizontal	12D	Orbital (traumatic)	Abducens palsy	Abducens palsy	Concordant
10	F/66	Rt	Vertical	2 M 5D	Infranuclear	IO or SR muscle palsy	IO palsy	Discordant
11	F/57	Rt	Vertical	3 M	Idiopathic	IO palsy	IO palsy	Concordant
12	M/44	Rt	Horizontal	15D	Infranuclear	Abducens palsy	Abducens palsy	Concordant
13	F/21	Rt	Vertical	3 M 10D	Infranuclear	Trochlear palsy	Trochlear palsy	Concordant
14	M/19	Lt	Vertical	1 M 25D	Supranuclear	SO palsy	SO palsy	Concordant
15	F/20	Rt	Vertical	5D	Infranuclear	Oculomotor palsy	Oculomotor palsy	Concordant

*CRT* computerized red glass test, *Rt* right, *Lt* left, *IO* inferior oblique muscle, *MR* medial rectus muscle, *SO* superior oblique muscle, *SR* superior rectus muscle

### Comparison between the CRT and the Hess test

**Fig. 2:**
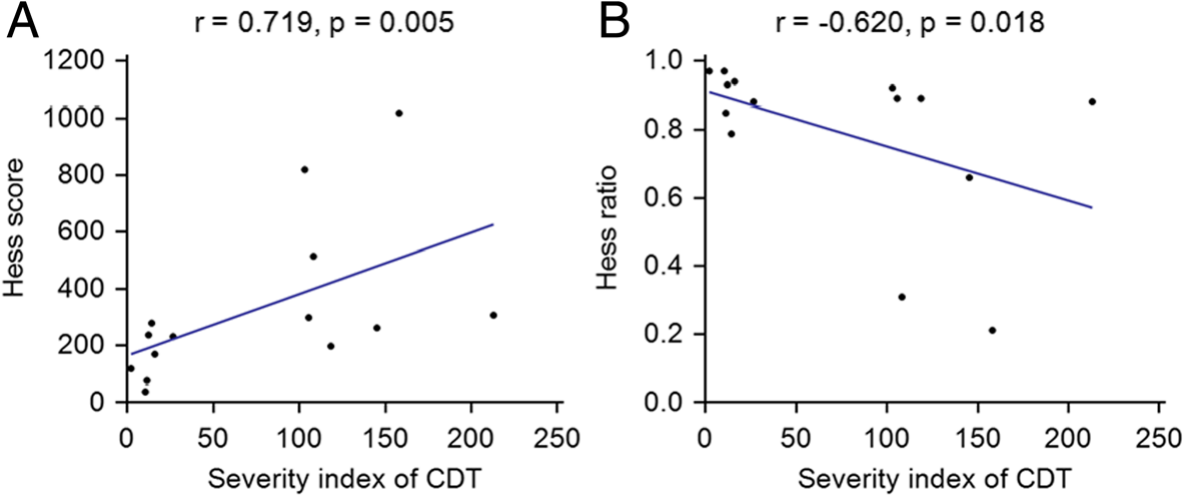
Correlation of the diplopia severity index with the Hess test. The degree of diplopia measured by the computerized red glass test (CRT) was correlated with the results of the Hess score (*r* = 0.719, *p* = 0.005) (**a**) and the Hess area ratio (*r* = −0.620, *p* = 0.018) (**b**)

**Fig. 3:**
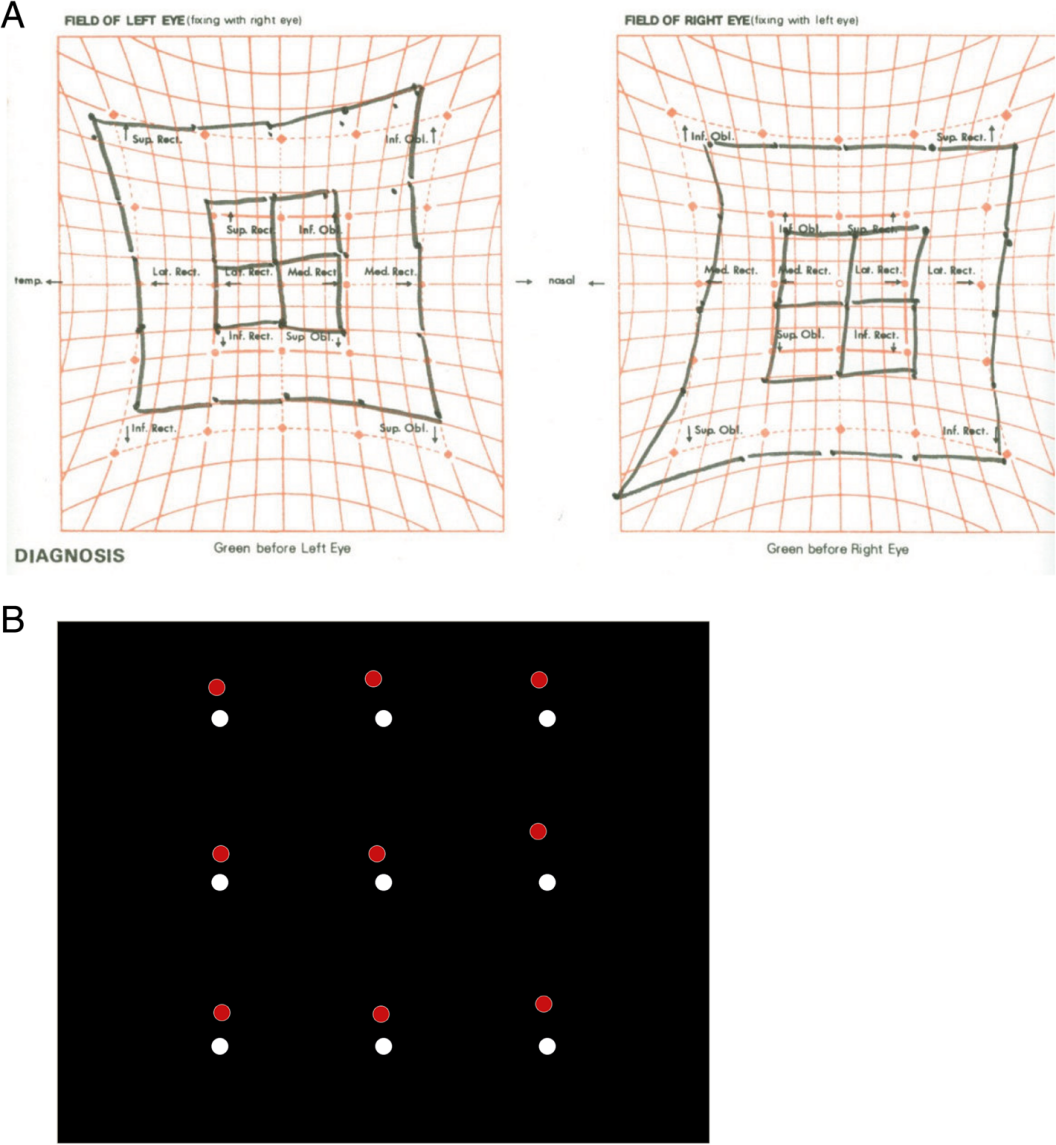
Discordant case between the CRT and the Hess test. The patient 10 showed the discordant result. The Hess test was indicated the presence of the inferior oblique palsy (**a**), whereas the computerized red glass test (CRT) showed patterns of both the inferior oblique and the superior rectus palsy (**b**)

The test-retest reliability of the CRT was excellent. During the three trials of the CRT, the median distances between the red dots and the center dots were consistent (overall ICC = 0.948, 95% CI 0.939–0.956). The degree of diplopia measured by the CRT was 52.07 (IQR 12.27–86.98). The median Hess score was 248 (IQR 175–304), and the HAR was 0.89 (IQR 0.81–0.93). The degree of diplopia from the CRT correlated well with both the Hess score (*r* = 0.719, *p* = 0.005) and the HAR (*r* = −0.620, *p* = 0.018) (Fig. 2). Weakened extraocular muscle assessed by the CRT were concordant with that of the Hess test in 13 (89.3%) out of 14 patients. The patient 10 showed the discordant result. The Hess test was interpreted to indicate the presence of the inferior oblique palsy, whereas the CRT showed patterns of both the inferior oblique and the superior rectus palsy (Fig. 3).

### Comparison between patients and controls

Compared to the healthy controls, the patient group showed a higher degree of deviation in the CRT. The median horizontal deviation of patients was 5.28 mm, and that of the healthy controls was 0.68 mm (*p* < 0.001). It was same in the median vertical deviation (4.17 mm for patients vs. 0.68 mm for controls, *p* = 0.003) and the median direct distance (18.87 mm for patients vs. 0.95 mm for controls, *p* < 0.001) (Table 2).

**Table 2:** Differences in degree of deviation between patients and controls

	Patients (*n* = 14)	Controls (*n* = 10)	*p* value
Horizontal deviation	5.28 (2.94-30.05)	0.68 (0.56-0.72)	<0.001
Vertical deviation	4.17 (2.39-18.28)	0.68 (0.63-0.74)	0.003
Direct distance	18.87 (5.39-42.88)	0.95 (0.76-1.10)	< 0.001

The values are expressed as the median (25 percentile-75 percentile)

## Discussion

In this pilot study, we demonstrated that the CRT was useful and feasible in the evaluation of binocular diplopia. The CRT can easily detect the presence of diplopia and provide quantitative values for the degree of diplopia. The test-retest reliability of the CRT was also excellent.

The red glass test is a commonly used bedside examination for the patients with diplopia. In the conventional red glass test, the patient was asked to see the light of penlight while a red filter or glass is placed over the right eye. Thus, the right eye sees a red light, and the left eye sees a white light. The test is performed in nine cardinal positions. When the action of a paretic muscle was examined, the red and the white dots are separated. The examiner asks the patient to point out where the images are most widely separated and to estimate their distance [3]. Although the red glass test is simple and helpful for rapid evaluation of binocular diplopia, the results depend solely on the patient’s response, and the location of target light is not standardized. These factors can bring poor inter−/intra-rater reliability.

To overcome those weaknesses of the red glass test, the CRT use a computer software. The CRT can collect and analyze the diplopia with consistency. Immediately after completion of the CRT, the program provides horizontal deviation, vertical deviation, direct distance, and the degree of diplopia. Along with data, the CRT also provides a summarized figure which is similar to the red glass test. Because the figure provides the binocular diplopic image as viewed by the patient in the nine cardinal positions, it enables examiners to easily identify weakened the extraocular muscles.

The Hess test is a gold standard test evaluating patients with diplopia. We demonstrated that the CRT is comparable with the Hess test in terms of localization and quantification. Compared to the Hess test, the CRT has several merits. First, the Hess test separately evaluates each eye. In contrast, the CRT provides binocular diplopic images according to the patients’ view to reflect the degree of diplopia in everyday life. Second, the CRT can be performed using a desktop computer and monitor in any place, including a stroke unit, an emergency room, or a clinic. Third, the elapsed time for the three CRT trials was less than 5 min, whereas a single trial of the Hess test requires twice as much time. Fourth, the CRT does not require a skilled technician and devices. By instructions, patients can easily understand the test and cooperate in the examination.

Although overall inter-rater and intra-rater reliability of the CRT were excellent, our trials had several limitations. First, the CRT may not be sensitive enough to detect subtle or severe diplopia. One of the study patients showed a discrepancy between the results of the Hess test and the CRT. In order to detect subtle or extensive diplopia, the Hess test might be useful. Second, head movements may influence the results. In fact, head movements can also affect the results of the Hess test. To minimize head movements, a neck collar was applied during the CRT. Third, the monitor size might have influenced the results. Further studies of testing with a smaller screen such as a laptop or a tablet, and a larger projector screen might be needed. Lastly, since this was a pilot study of CRT, further research with a larger sample size and follow-up study in the same patient would be needed.

## Conclusion

We demonstrated that the CRT was useful and feasible for the evaluation of diplopia. As we showed, the CRT can be used in any place with a desktop computer and a monitor. Because the CRT provides not only the quantitative data but also the qualitative figure, the CRT can be used in patients with diplopia to improve routine bedside examination.

## Abbreviations

CRT: Computerized red glass test
HAR: Hess area ratio
ICC: Intraclass correlation coefficient
IQR: Interquartile range

## References

[1] Rucker JC, Tomsak RL (2005). Binocular diplopia. A practical approach. Neurologist.

[2] Danchaivijitr C, Kennard C (2004). Diplopia and eye movement disorders. J Neurol Neurosurg Psychiatry.

[3] Blumenfeld H (2010). Neuroanatomy through clinical cases.

[4] Roodhooft JM (2007). Screen tests used to map out ocular deviations. Bull Soc Belge Ophtalmol.

[5] Nam HS, Park E, Heo JH (2013). Facilitating stroke management using modern information technology. J Stroke.

[6] Roper-Hall G (2006). The hess screen test. Am Orthopt J.

[7] Aylward GW, McCarry B, Kousoulides L, Lee JP, Fells P (1992). A scoring method for Hess charts. Eye (Lond).

[8] Furuta M, Yago K, Iida T (2006). Correlation between ocular motility and evaluation of computed tomography in orbital blowout fracture. Am J Ophthalmol.

